# The Motivation-Based Promotion of Proactive Control: The Role of Salience Network

**DOI:** 10.3389/fnhum.2018.00328

**Published:** 2018-08-14

**Authors:** Lei Qiao, Lei Xu, Xianwei Che, Lijie Zhang, Yadan Li, Gui Xue, Hong Li, Antao Chen

**Affiliations:** ^1^College of Psychology and Sociology, Shenzhen University, Shenzhen, China; ^2^Key Laboratory of Cognition and Personality of Ministry of Education, Faculty of Psychology, Southwest University, Chongqing, China; ^3^Key Laboratory for Neuroinformation, Center for Information in Medicine, University of Electronic Science and Technology of China, Chengdu, China; ^4^Monash Alfred Psychiatry Research Centre, The Alfred and Central Clinical School, Monash University, Melbourne, VIC, Australia; ^5^MOE Key Laboratory of Modern Teaching Technology, Shaanxi Normal University, Xi’an, China; ^6^State Key Laboratory of Cognitive Neuroscience and Learning & IDG/McGovern Institute for Brain Research, Beijing Normal University, Beijing, China

**Keywords:** dual-mechanism of control, independent component analysis, AX-Continuous Performance Task, salience network, proactive control, reactive control, reward

## Abstract

It has been shown that reward motivation can facilitate proactive control, a cognitive control mode that is characterized of prior preparation and sustained holding of the goal-relevant information in working memory. However, it remains to be established the neural networks that may be involved in this promotion effect. In this study, participants underwent the AX-Continuous Performance Task (AX-CPT) that measures relative proactive control during functional magnetic resonance imaging (fMRI) scanning. We employed independent component analysis to decompose multiple brain networks and identified the task related network. Results showed that the salience network (SN) was engaged in the AX-CPT protocol. Importantly, our data demonstrated that reward modulated the association between task engagement of SN and proactive control, whereby the positive correlation was particularly observed in the reward condition. Moreover, reward modulated task engagement of the SN in a proactive manner, which may contribute to the behavioral proactive performance. Overall, our data suggest the involvement of SN in the reward facilitation effect of proactive control.

## Introduction

Cognitive control is defined as a set of processes that allow individuals to flexibly coordinate thoughts and behaviors in accordance with internal goals (Miller and Cohen, 2001). It has been widely studied how cognitive control influences our behavior and daily life. Among these studies, a theoretical framework—the dual-mechanism of control (DMC) theory—postulates that there are two distinct cognitive modes in cognitive processing, namely, reactive control and proactive control (Braver et al., 2007; Braver, 2012). In reactive control, task-relevant information (e.g., task instructions, goals, stimulus-response mapping) is processed in a transient manner (Braver, 2012). Reactive control is helpful to detect and resolve interference only when an interference occurs; thus, attention is recruited as a late correction mechanism. Meanwhile, in proactive control, task-relevant information is actively maintained in working memory to direct attention and possible responses. Proactive control is a sustained form of control, which can be engaged prior to the presentation of stimuli and can promote rapid and efficient responses (Jaspar et al., 2014; Chiew and Braver, 2016).

Previous studies have suggested that reward motivation can facilitate goal-directed behavior via the adoption of an optimal cognitive strategy (Pochon et al., 2002; Gilbert and Fiez, 2004; Taylor et al., 2004; Small et al., 2005; Fröber and Dreisbach, 2014; Etzel et al., 2015). Indeed, the DMC theory proposes that reward motivation can reliably enhance a proactive control mode relative to reactive control. For instance, Locke and Braver (2008) compared reward and penalty effects on strategy transformation between proactive and reactive control. Results demonstrated that reward condition showed sustained activity of proactive control, whereby the penalty condition produced a shift toward a more reactive, probe-based pattern of activation. Jimura et al. (2010) also found that reward can promote proactive control, and that individuals with high reward sensitivity exhibited better working memory performance in rewarding contexts.

In the context of motivation and cognitive control, it is interesting to investigate how the brain networks may interact to support the motivational effects in control (Botvinick and Braver, 2015). Although the literature supports the idea that reward motivation can promote a proactive mode of cognitive control, little is known about the activity of neural networks that may underlie this process. A network perspective may provide insights of the synchronized brain activity and the dynamic information changes between brain regions that support the cognitive control modes (Papo et al., 2014). According to the literature, it is hypothesized that the fronto-parietal network (FPN) and/or the salience network (SN) would be involved in the incentive enhancement of proactive control as of their extensive involvement in reward and cognitive control.

The FPN, mainly composed of the lateral prefrontal cortex (lateral PFC) and the posterior parietal cortex (PPC), plays an important role in both cognitive control and reward. The FPN is engaged in various cognitive tasks in which cognitive control is needed to guide selective attention (Dosenbach et al., 2007, 2008; Cole et al., 2013). Meanwhile, reward motivation has been reported to enhance task coding in fronto-parietal cortex. Specifically, the authors trained a multi-voxel classifier in baseline session and used this classifier to distinguish different task sets in a reward session. They found that the classification accuracy was higher in reward trials than non-reward trials in fronto-parietal regions, and that the enhancement of the decoding accuracy mediated the improvement of behavioral performance (Etzel et al., 2015). Importantly, it has been suggested that the lateral PFC also plays an important role in the motivation-based enhancement of proactive control, in which proactive control was associated with sustained activity of the lateral PFC (Locke and Braver, 2008; Costumero et al., 2015).

Similarly, the SN is also involved in both cognitive control and reward. The SN is known to support the detection of behaviorally relevant stimuli (Uddin, 2014; Dajani and Uddin, 2015), the maintenance and implementation of task sets (Dosenbach et al., 2006; Nelson et al., 2010), and the organization of behavioral responses (Medford and Critchley, 2010). Moreover, the SN can initiate cognitive functions by sending control signals to other large-scale networks that are associated with the allocation of attention and working memory resources (Menon, 2011). Meanwhile, the amygdala and the substantia nigra/ventral tegmental area (SNc/VTA), two subcortical structures of the SN, are involved in detecting emotional and reward saliency (Seeley et al., 2007; Menon, 2011).

In summary, this study aimed to examine the role of the synchronizing activity in the FPN and/or SN in the motivation-based facilitation of proactive control. To this end, we hypothesized that these two networks would show the following features: (1) FPN/SN may be engaged in the proactive control task (i.e., AX-Continuous Performance Task, AX-CPT) whereby the activity of these two network is associated with the task phase and task performance; (2) task engagement of FPN/SN may be associated with proactive control index which was measured by the relative performance of BX and AY trials; and (3) reward may modulate the association between FPN/SN engagement and proactive control. Specifically, we assumed that reward might promote the relationship between FPN/SN engagement and proactive control. Moreover, previous studies have suggested that the effect of reward motivation on cognitive control can be reflected in the interaction between two large-scale brain networks. These include one brain network representing the reward value and another performing specific cognitive functions (Botvinick and Braver, 2015). Based on the proposed functioning of SN (i.e., detection of reward saliency and translation of control signals to other brain networks) and FPN (i.e., motivation-based enhancement of proactive control), we propose that SN activity may represent the reward value and signal FPN to implement specific control functions. We therefore hypothesized that: (1) reward may modulate the relationship between SN and FPN activity (or connectivity between these two networks), making them more correlated in the reward condition; and (2) FPN activation may mediate the positive relationship between SN activity and proactive control. That is, SN activity may enhance the activation of the FPN, which in turn promotes proactive control.

## Materials and Methods

### Participants

Twenty-four right-handed, healthy young adults participated in this study. All of them had normal or corrected-to-normal vision, and none of them had a history of neurological or psychiatric illness. Two volunteers were excluded, as they did not complete the testing. In addition, another two subjects were excluded as their overall head motion was above 2 mm in translation or 2° in rotation during scanning. In total, 20 subjects were included in the subsequent analysis, which consists of nine females (mean = 21.7 years, SD = 1.8) and 11 males (mean = 21.6 years, SD = 1.4). The study was approved by the Southwest University Brain Imaging Center Institutional Review Board. All subjects gave written informed consent in accordance with the Declaration of Helsinki.

### Task and Procedure

Participants performed the reward version of the AX-CPT in a block design. The AX-CPT has been widely used to investigate the dynamic changes between proactive and reactive control (Braver, 2012). In the classical AX-CPT, participants need to respond to a probe (X/Y) which follows a cue (A/B). “B” represents any non-A letters and “Y” stands for any non-X letters. Only the A-cue followed by the X-probe requires a target response, whereas all other combinations (A-Y, B-X, B-Y) require a non-target response. In this study, the cue letter was either A or B, and the probe letter was either X or Y. Participants were asked to respond to the target (AX combination) with the right index finger, and respond to the non-target with the left index finger (AY, BX, BY). Because the A-X combination occurs at a high frequency (70%), both the A-cue and the X-probe could produce high target response bias. However, the inconsistent response tendency between the cue and the probe in the A-Y and the B-X combinations would cause interference, participants thus need to switch between proactive and reactive control strategies to achieve better task performance. Specifically, a proactive control strategy helps resolve the conflict in the B-X combination, but a reactive control strategy is useful for resolving the interference in the A-Y combination (Locke and Braver, 2008; Chatham et al., 2009). Thus, task performance in the AY and BX trials can be considered as an index of the reactive and proactive control strategy respectively. Here, we calculated proactive control index for both RT and error rate using the behavioral shift index (BSI: [AY − BX]/[AY + BX]), which measures to what extent subjects tend to adopt the proactive control strategy (Paxton et al., 2006, 2008; Chiew and Braver, 2014). A smaller value of this index means less proactive and more reactive control, while a larger value means more proactive and less reactive control (Lamm et al., 2013; Zhang et al., 2015; Maraver et al., 2016).

In the reward context, participants were informed that they would be rewarded for their quick and correct response if the prime stimulus of that trial is “$$$,” but not if the prime is “###.” In the baseline context, neither incentives nor the meaning of the motivation cues were provided. Thus, the feedback “+50” was only presented in the reward context which means that participants would receive 50 cents, and “+--” was presented in both the reward and baseline context indicating that no reward was provided. There were two runs, i.e., a baseline and a reward run in the task, and each run has three blocks. The baseline run was followed by the reward run. The sequence was fixed because reward was only provided if the response in the reward block was correct and faster than the average reaction time (RT) in the baseline block (Locke and Braver, 2008; Chiew and Braver, 2013). Each block included 40 trials, with 28 AX trials (70%), 4 AY trials (10%), 4 BX trials (10%) and 4 BY trials (10%). These trials were presented in a pseudorandom order and each block lasted for 9 min. A 10-s rest was set at the beginning of the experiment, and the imaging data in this period were abandoned so that the scanner could reach a steady state. One practice block was conducted before the actual testing. The actual task started if the accuracy in the practice block reached at least 90%.

**Figure 1:**
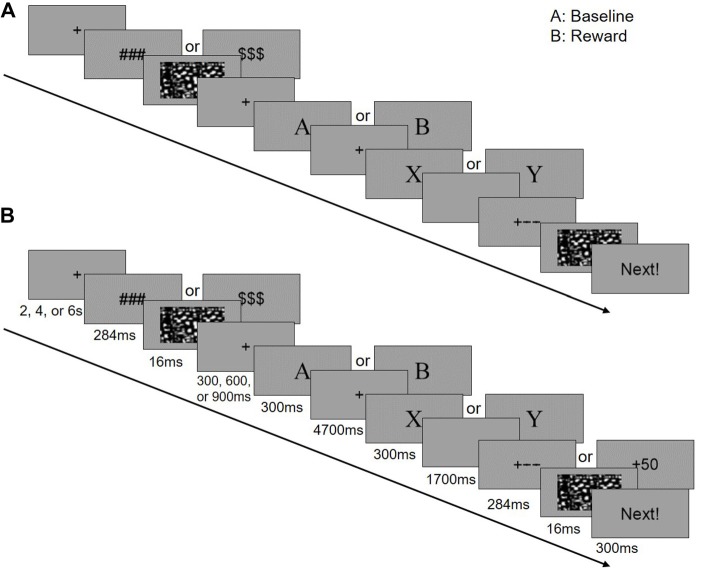
Experimental design of this study. In the scanner, the participants performed a block designed AX-Continuous Performance Task (AX-CPT). There are two runs in the task (a baseline and a reward run), and each run has three blocks (40 trials in each block). The baseline run was followed by the reward run. **(A)** Single trial for the baseline context. **(B)** Single trial for the reward context.

An illustration of the task is provided in Figure 1. The letters of the cue and target stimuli were presented at the center of the screen in sequence in 36-point size, Times New Roman font. A random jitter (fixation, 2 s, 4 s, or 6 s) was set for the inter-trial interval (ITI). Then a reward symbol (“$$$” or “###”) and the following mask were presented for 300 ms. Next, a random jitter fixation screen (300 ms, 600 ms, or 900 ms) was shown before the cue screen (300 ms), followed by a delay period (4,700 ms), a target screen (300 ms) and a blank screen for response (1,700 ms). The timing for response was fixed to 2,000 ms and not terminated by a response. Then, a feedback with the following mask screen (300 ms) were displayed. In the end of each trial, a reminder for the next trial “Next!” was presented for 300 ms.

### fMRI Data Collection

All functional magnetic resonance imaging (fMRI) were collected on a Siemens 3T Trio scanner (Siemens Medical Systems, Erlangen, Germany). A foam pad was used to minimize the subjects’ head motion. fMRI images were acquired by using the gradient-echo echo planar imaging (GRE-EPI) sequence: TR/TE = 2,000 ms/30 ms; flip angle = 90°; resolution matrix = 64 × 64; FOV = 220 × 220 mm^2^; thickness = 4 mm; and acquisition voxel size = 3.4 × 3.4 × 4 mm^3^. We used 32 slices to cover the entire brain.

### Data Preprocessing

fMRI data were preprocessed using statistical parametric mapping 8 (SPM8, Welcome Trust Centre for Neuroimaging, London, UK^1^) implemented on a MATLAB 2009b (Math Works, Natick, MA, USA) platform. For each block, the first 5 functional volumes were discarded. The remaining scans were slice-time corrected and subsequently realigned to the first image to correct for head motion. Subsequently, all realigned images were spatially normalized to the Montreal Neurological Institute (MNI) template and resampled into 3 × 3 × 3 mm^3^. Thereafter, the images were smoothed with a 6 mm full-width at half-maximum (FWHM) Gaussian kernel to increase the signal-to-noise ratio (SNR).

### Behavioral Data Analysis

We first calculated the relative proactive control index for both RT and error rate using the formula [(AY − BX)/(AY + BX)] (Paxton et al., 2006, 2008; Chiew and Braver, 2014). This proactive index is a standardized measure that ranges from −1 to +1. There were three levels of incentive in this study, namely, the baseline trials, non-incentive trials in the reward context and incentive trials in the reward context. To examine the reward effect on proactive control, baseline trials and incentive trials in the reward block were examined with paired sample *t*-tests. The ANOVA analysis between reward prime (### vs. $$$) and block (baseline vs. reward block) was not presented here as we were not interested in the main or the interaction effect between these two variable. Moreover, we did not find any significant result of the main or the interaction effect between reward prime and block.

### Independent Component Analysis of fMRI Data

Functional imaging data can be decomposed to brain networks using independent component analysis (ICA), which has been increasingly applied to task-fMRI data (Xu et al., 2013a, 2016; Yip et al., 2018). ICA is a data-driven method to characterize the temporal coherence of neural activities and functional integration of neural networks (Meda et al., 2012; Smith et al., 2012). Meanwhile, cognitive task performance involves the recruitment of multiple brain regions, it is plausible that reward may modulate the functional integration between these various task related networks, rather than a single region. Moreover, ICA is sensitive to neural activity patterns that may not be detected by traditional whole-brain analysis based on general linear model (GLM), which has been suggested to be able to provide supplementary information to GLM-based methods (Xu et al., 2016; Yip et al., 2018). Furthermore, in this study, we are interested in understanding the functional synchronization (connectivity) of the brain regions or networks, which provides a context for ICA to fit in.

ICA was performed using the MATLAB platform group ICA toolbox (GIFT^2^, version 3.0a), which can identify spatially independent and temporally coherent networks. Preprocessed fMRI data were submitted to principal component analysis to reduce the dimensions for each subject. Then group spatial ICA was conducted with the Infomax algorithm (Bell and Sejnowski, 1995), and the number of component in our data was estimated to be 24 using minimum description length criteria in the software (Li et al., 2007). Group spatial ICA generates spatial maps and time series of bold signals for each component. The spatial maps characterize the brain regions included in the network, with values in these maps representing the temporal synchronization between the time series of a given voxel and a given network, reflecting how strongly each voxel is coupled with a given network (Allen et al., 2012). Each subject’s time courses and spatial maps were then back-reconstructed by using the data from the data reduction procedure. In total, 24 spatial maps and related time series were produced for each participant. We can carry out group-level random effect hypothesis tests to compare individual differences or condition differences in each spatial map or component time series. The analysis of spatial maps allows us to compare the group difference in the intensity of functional connectivity, while the analysis of the temporal process information enables us to judge whether the participants engaged in the fMRI task or not (Meda et al., 2009).

In the context of task-fMRI, some components may reflect networks that are helpful in performing tasks, while other components may represent unrelated intrinsically connected networks or spatiotemporal sources of noise (Hallquist et al., 2018). To identify the task-related network, we performed regression analysis between component time series and stimulus events convolved with a canonical hemodynamic response function (HRF), which is usually applied in ICA of task-fMRI data (e.g., Zhang and Li, 2012). Similar to conventional voxel wise GLM analyses, the resulting regression coefficients (beta values) represent the influence (correlation) of the stimulus on BOLD activity (Kim et al., 2009). The main difference between ICA and single voxel analysis is that component time courses represent a group of temporally coherent activities in functional coupling regions. Therefore, like voxel wise GLM regression coefficient, the correlation between component time courses and task phase reflects both the intensity of task-related network activity and the temporal accordance of the network to stimulus presentation (Hallquist et al., 2018). Thus, an increase or decrease in regression coefficient (beta values) in one condition relative to another represents an increase or decrease in the task-related activity of the corresponding network (Xu et al., 2013b).

### Identification of the Task Related Network

**Figure 2:**
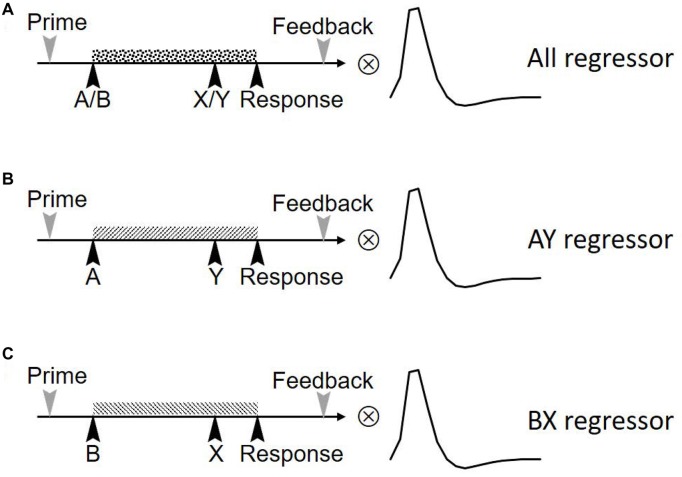
Three regression factors for the task in the study. Three independent boxcar-shaped regression factors were set up. These three sequences represent the regression factors for **(A)** all trials (including AX, AY, BX, BY), **(B)** AY trials and **(C)** BX trials. The three sequences were convolved with statistical parametric mapping’s (SPM’s) hemodynamic response function (HRF) to form the final task sequence.

We first examined if the FPN/SN activation is associated with the task phase of the AX-CPT as well as the task performance. Three separate boxcar regressors were first defined by a train of cue onsets with a duration of 6 s (form cue onset to the blank screen waiting for response to include 3TR), representing all trials (including AX, AY, BX, BY), AY trials and BX trials, respectively (Figure 2). These regressors were then convolved with a canonical HRF. Temporal regression was then performed between the time courses of the 24 components and three task regressors. By doing this we can estimate the association (beta weights) between the component time series and the three task regressors. The beta values represent the degree of synchrony between the components’ time courses and the task phase, which indicates whether the network was engaged during this task (Meda et al., 2009). An increase or decrease in beta values in one condition relative to another represents an increase or decrease in the task-related activity of the corresponding network (Xu et al., 2013b). We first visually checked the most correlated network, than we performed a spatial correlation between a predefined SN mask^3^ (Shirer et al., 2012) and the identified components. This template included the main SN regions such as the bilateral anterior insula (AIns) and the dorsal anterior cingulate (dACC; Seeley et al., 2007). To further examine whether or not FPN and SN are involved in the task, we calculated the correlation between network activity (beta values) and behavioral performance for both RT and ACC, separately.

### The Association Among Reward, AY/BX Engagement of the Salience Network and Proactive Control

Since we found SN but not FPN is involved in the task, the following analysis was only performed on the SN. We first calculated the association between AY/BX engagement of the SN and proactive control, with the assumption that SN engagement in AY/BX trials may predict the behavioral proactive control index. Considering that better performance on BX trials indicates proactive control, and that better performance on AY trials indicates reactive control (Chatham et al., 2009), higher level of BX-AY engagement would be associated with better proactive control.

Subsequently, we explored whether reward could modulate the association between AY/BX engagement of the SN and proactive control. To this end, the correlation coefficients between BX-AY engagement of the SN and proactive control of the baseline and reward condition were calculated, respectively. Then, Fisher’s *r*-to-*z* transformation was performed to increase the normality of the correlation coefficients. Finally, *z* values of correlation coefficients were subjected to significant tests.

### The Correlation Among Reward, Trial Types and AY/BX Engagement of the Salience Network

We further explored why reward might modulate the association between AY/BX engagement of the SN and proactive control. As the relationship between BX-AY engagement of the SN and proactive control might be greater in the reward condition, we assumed that reward might enhance task engagement of SN proactively. In other words, compared to baseline, reward might enhance BX engagement more than AY engagement of the SN. This proactive neural network engagement may support the behavioral proactive performance. We thus submitted the AY/BX engagements to repeated measure ANOVA with trial types (AY vs. BX) and reward (reward vs. baseline) as within-subject factors to determine if the interaction between trial types and reward is significant.

## Results

### Behavioral Results

**Figure 3:**
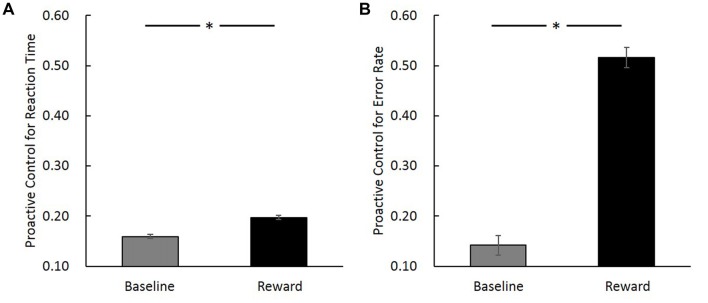
Proactive control index ([AY − BX]/[AY + BX]) in baseline and reward block (reward trials in the incentive block) for reaction time (RT; **A**) and error rate **(B)**. The horizontal axis denotes different conditions (baseline vs. reward) and the vertical axis represents the magnitude of proactive control (error bars denote standard errors). *Indicates *p* < 0.05.

To validate the effect that reward can modulate proactive control, a paired sample *t*-test was performed to compare the proactive control of baseline trials with incentive trials in reward block. Results revealed that incentive trials in reward block showed higher proactive control for both RT (*t*_(19)_ = 2.44, *p* = 0.02) and error rate (*t*_(19)_ = 3.07, *p* = 0.01; Figure 3). This result is consistent with previous studies showing that reward can facilitate proactive control (Locke and Braver, 2008; Jimura et al., 2010; but see Boehler et al., 2014).

### The Salience Network Is Engaged in the AX-CPT

Visual inspection suggested that the most positively correlated network with the task was SN (*t*_(19)_ = 10.73, *p* = 1.67 × 10^−9^). Regions, *T* values, and MNI coordinates of this network were listed in Table 1. Spatial correlation results confirmed the relationship in which this component showed the highest positive spatial correlation with the SN template (0.57). We also submitted a FPN template^4^ for spatial correlation and identified the component that showed the highest spatial correlation (0.45). However, this component was not correlated with the task (*p* > 0.1).

**Table 1:** Regions of the salience network (SN) that are correlated with the task phase of AX-CPT.

Regions	Voxels (n)	*T*	MNI coordinates		
			*x*	*y*	*z*
SMA/dACC	721	10.8	9	8	49
L. AIns	137	8.1	−30	23	4
R. AIns	170	10.8	36	17	4
L. MFG	121	6.8	−30	50	19
R. MFG	103	5.8	30	50	25

*Corrected at p < 0.01. AX-CPT, AX-Continuous Performance Task; SMA, supplementary motor area; dACC, dorsal anterior cingulate; AIns, anterior insula; MFG, middle frontal gyrus; L, Left; R, right*.

**Figure 4:**
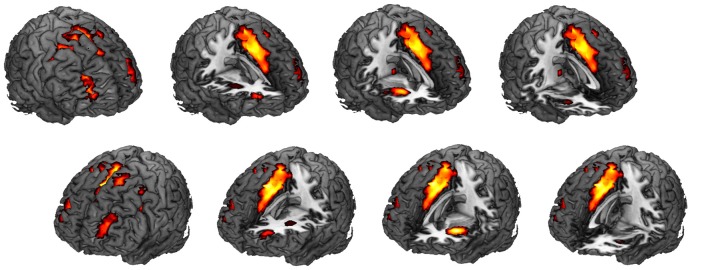
Spatial map for the component that was most correlated with the task. One sample *t*-test was performed for this spatial map (corrected for multiple comparisons with the FDR, *p* < 0.01). See Table 1 for specific regions, *T* values and montreal neurological institute (MNI) coordinates. This component showed the highest positive spatial correlation (0.57) with an SN template. FDR, false discovery rate; SN, salience network.

**Figure 5:**
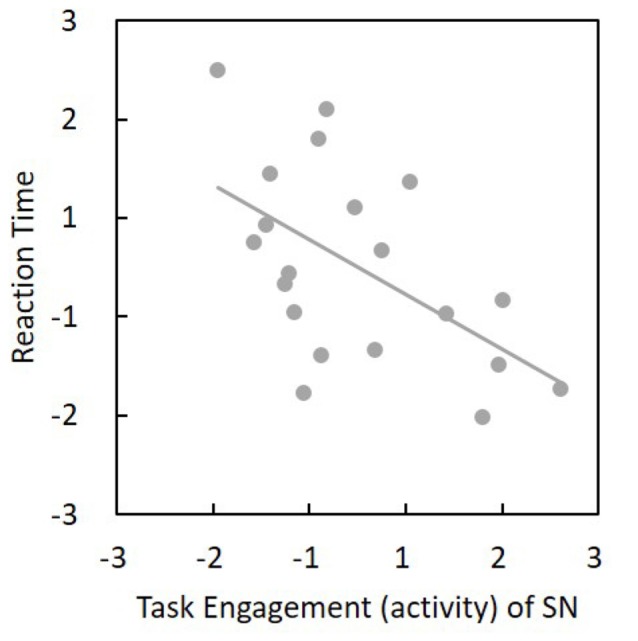
Task engagement (activity) of SN was negatively associated with reaction time (*r*_(18)_ = −0.55, *p* = 0.02). The horizontal and vertical values represent standardized *z*-scores. Thus, a more task-engaged SN would show a faster reaction. SN, salience network.

Among the 24 components, we found that SN activity is associated with the AX-CPT task. One-sample *t*-tests were applied to the beta values of the SN to verify that the SN time series was significantly (*t*_(19)_ = 10.73, *p* = 1.67 × 10^−9^) associated with the task course and was thus task-engaged (Figure 4). To be more confirmative, the correlations between the task engagement of the SN and the behavioral data (RT and error rate) were examined. A more task-engaged SN was associated with a faster RT (*r*_(18)_ = −0.55, *p* = 0.02; Figure 5) but not for error rate (*r*_(18)_ = −0.36, *p* = 0.12), which indicates that the SN is involved in the AX-CPT task. The deeper SN engaged in the task, the faster RT would show. Meanwhile, the activity of FPN was not correlated with either RT (*r*_(18)_ = −0.07, *p* = 0.078) or error rate (*r*_(18)_ = −0.004, *p* = 0.99). To avoid possible false positive results, multiple comparison correction was applied to our results by setting false discovery rate (FDR) to <0.05 (Benjamini and Hochberg, 1995; Benjamini and Yekutieli, 2001).

### Reward Enhances the Correlation Between AY/BX Engagement of the Salience Network, and Proactive Control

**Figure 6:**
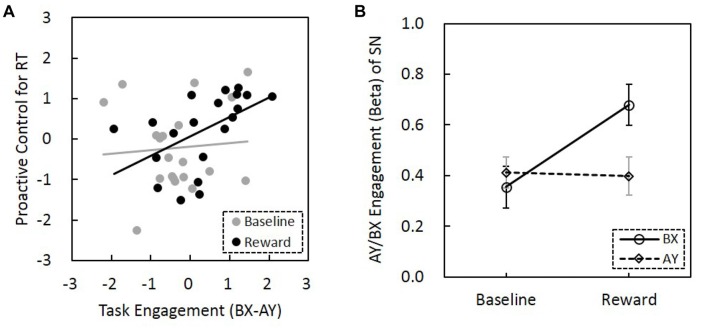
Relationship among reward motivation, task engagement of SN and proactive control. **(A)** Compared to the baseline, reward motivation increased the association between task engagement (activity) of SN (BX-AY) and proactive control. Before the comparison, Fisher’s r-to-z transformation was performed to increase the normality of the correlation coefficients, and *z* values of correlation coefficients were subjected to paired sample *t*-test (baseline vs. reward). The horizontal and vertical values represent standardized *z*-scores. **(B)** The interaction between reward and trial types was significant, suggesting reward differentially influenced the SN engagement for AY and BX trials (error bars denote standard errors). SN, salience network.

The correlation between BX-AY engagement of the SN and proactive control index for both RT and error rate were calculated, respectively. We found that BX-AY engagement of the SN, which may reflect the level that SN is proactively engaged, is positively associated with proactive control index for RT (*r*_(18)_ = 0.43, *p* = 0.056), but not for error rate (*r*_(18)_ = 0.24, *p* = 0.32). Greater difference between BX and AY engagement was associated with better proactive control. Further analysis revealed that this positive correlation was only present in the reward condition (*r*_(18)_ = 0.51, *p* = 0.02), but not for the baseline (*r*_(18)_ = 0.08, *p* = 0.75). Moreover, the difference between these two correlation coefficients was significant (*Z* = 5.9, *p* = 1.79 × 10^−9^; Figure 6A). Thus, reward enhanced the correlation between BX-AY engagement of the SN and proactive control, making them positively correlated.

### Reward Modulate the AY/BX Engagement of the Salience Network

The ANOVA for the AY/BX engagement of the SN found a significant main effect of reward (*F*_(1,19)_ = 5.66, *p* = 0.021; Figure 6B). SN engagement was higher for the AY and BX trials in the reward condition than in the no-reward condition. The effect of trial type was not significant (*F*_(1,19)_ = 2.5, *p* = 0.12). Importantly, the interaction between reward and trial types was significant (*F*_(1,19)_ = 4.61, *p* = 0.036), that is, reward can promote significantly greater task engagement of SN in trials that need proactive control (BX trials) than trials that need reactive control (AY trials). Thus, reward might enhance task engagement of SN proactively, and this proactive neural network engagement might support the behavioral proactive performance.

## Discussion

The present study investigated the relationship among reward, task engagement of the SN, and proactive control. Consistent with previous studies (Locke and Braver, 2008; Jimura et al., 2010), our results demonstrated that reward motivation could enhance the proactive mode of cognitive control. We further found that the SN is involved in the AX-CPT task, in which the time series of SN is positively correlated with task regressor, and that the task engagement of SN is positively associated with behavioral performance. Interestingly, our data demonstrated that reward could modulate the association between the SN engagement and proactive control whereby the SN engagement is positively associated with proactive control particularly in the reward condition. Furthermore, we found a significant interaction effect between reward and trial types, indicating that reward enhances greater task engagement of SN in trials that need proactive control (BX trials) than trials that need reactive control (AY trials). However, it is possible that the different responses between A and B cue may account for some of the results. Therefore, it is purposed that the SN network may be more engaged in B cue compared to A cue, suggesting increased utilization of contextual cues (proactive control) as B cues are 100% valid for preparing a non-target response (Braem et al., 2014; Fröber and Dreisbach, 2014; Hefer and Dreisbach, 2017).

We explored the control mode shift (tendency) toward relative proactive control, as indicated by the BSI ([AY − BX]/[AY + BX]). A smaller value of this index means less proactive and more reactive control, while a larger value means more proactive and less reactive control (Lamm et al., 2013; Zhang et al., 2015; Maraver et al., 2016). In this regard, we assume that reward may decrease reactive control, since task performance in AY and BX trials can be considered as an index of the reactive and proactive control strategy, respectively (Braver et al., 2009; Polizzotto et al., 2018). Therefore, higher value of the BSI in the reward condition would also imply relatively lower reactive control in AX-CPT. It is reported that reward can enhance the task coding (Etzel et al., 2015), increase the maintenance of goal-relevant information (Zedelius et al., 2011), and promote cognitive stability (Fröber and Dreisbach, 2016; Hefer and Dreisbach, 2016, 2017; Fröber et al., 2018). In the reward condition of AX-CPT, the coding (representation) of A/B-cue and the corresponding response tendencies may be increased, which was also more stably maintained in the following period until response was made. Therefore, the A-cue would induce and stably maintain a stronger target response tendency, which would decrease the flexibility and detrimental for AY trials (non-target). While the B-cue would induce and stably maintain a stronger non-target response tendency, which is beneficial for BX trials (non-target; Hefer and Dreisbach, 2017; Yee and Braver, 2018). Thus, reward may increase proactive control and decrease reactive control in AX-CPT.

In this study, we found that the SN activity is associated with the AX-CPT task. SN is widely involved in cognitive control processes, e.g., conflict monitoring (Kerns et al., 2004), salience processing (Seeley et al., 2007), interference resolution (Nee et al., 2007), the maintenance and implementation of task sets (Dosenbach et al., 2006; Nelson et al., 2010), and the organization of behavioral responses (Medford and Critchley, 2010). Individuals need to flexibly adjust the proactive and reactive control strategies to solve the conflict in AY and BX trials in this protocol. Moreover, each trial type of AY, BX and BY only covers 10% of all trials. Thus, these trials may be considered as salience or oddball stimuli. In addition, the delay period between the cue and the probe requires the maintenance of goal-related information in working memory. In this regard, it is not surprising that the SN is engaged in AX-CPT, which could measure relative proactive control. The higher activation of the SN in the task, the better the task performance.

Our data demonstrated that reward could modulate the association between the SN engagement and proactive control. Previous studies have shown that SN is involved in reward-based decision making (Kennerley et al., 2011; Botvinick and Braver, 2015). Moreover, dACC, a core node of SN, plays a key role in the association between reward and cognitive control. Specifically, the expected values of control are suggested to be computed in the dACC, which then selects appropriate control functions based on the expected values. These cognitive controls are subsequently implemented in the sub-regions of the lateral PFC (Shenhav et al., 2013, 2016). Moreover, the SN is structurally connected with the subcortical and limbic structures such as the amygdala and SNc/VTA, which are involved in the processing of reward and motivation (Menon and Uddin, 2010; García-García et al., 2013). Importantly, SN includes regions (e.g., dACC and AIns) that are responsive for interoceptive-autonomic, conflict monitoring and reward-processing (Seeley et al., 2007). Information from these regions may thus be integrated by SN to relay the signal to other brain regions or organize a response. Therefore, reward motivation in this study may result in enhanced reward coding in the SN, which is associated with relatively more proactive control.

Importantly, our data demonstrated that reward could modulate the AY/BX engagement of the SN proactively, resulting in greater improvement of BX engagement than AY engagement. This neural modulation of the SN activity may account for the behavioral proactive performance after reward, since both ACC and the dopamine (DA; a neurotransmitter that is important for reward, motivation and cognitive control function) system can link reward with cognitive control (Botvinick and Braver, 2015; Westbrook and Braver, 2016). Moreover, phasic activity of DA neurons was found in response to reward cues (Schultz, 1998), while tonic DA release was associated with sustained motivational behaviors (Howe et al., 2013). In fact, the phasic and tonic DA signals not only support the processing of reward and motivational information (Niv, 2007), but are also involved in the modulation of proactive control (Cohen et al., 2002). Thus, it may be the case that reward increases the striatal DA release, which then modulates task engagement of the SN and promotes the persistence cognitive control (Westbrook and Braver, 2016).

Surprisingly, we found that the FPN is not involved in the task. It seems counter-intuitive since lateral PFC as one of the core regions of FPN has been commonly activated in motivation–cognitive control interaction studies (e.g., Bahlmann et al., 2015). However, it is possible that a single region (lateral PFC) may not be sufficient to represent FPN, as part of the lateral PFC may also belong to SN. The SN identified in this study includes portion of the lateral PFC, though the core regions of SN were AIns and dACC (Figure 4 and Table 1). Therefore, SN but not FPN in our result may be compatible with previous studies. It has been suggested that BOLD signal in the SN comes earlier than the FPN, and that activity of the SN may modulate FPN activity. For instance, a number of studies suggested that the SN plays a causal role in the switching between the FPN and the default mode network (e.g., Sridharan et al., 2008; Goulden et al., 2014). The dACC as a key node in SN was also found to signal the FPN node (dorsolateral PFC) for top-down control (Botvinick et al., 2001; Kerns et al., 2004; Liston et al., 2006). Therefore, SN activation may be necessary to initiate the functioning of the FPN. In line with this idea, prior studies have indicated that the AIns (one core region of the SN) contributes to the generation of control signals that are critical for the “stable maintenance of task mode and strategy” (Dosenbach et al., 2007). Menon and Uddin (2010) further assumed that the AIns is involved in the detection of salient stimuli. When a salient stimulus is detected, the AIns initiates control signals that are then sustained by the ACC and the lateral PFC. Similarly, it is proposed that cognitive control includes three major components, namely, monitoring, specification and regulation (Shenhav et al., 2013, 2016). The dACC monitors the current circumstances and notifies the lateral PFC which task should be undertaken, while the lateral PFC has the capacity to conduct or regulate lower-level information processing (Shenhav et al., 2013, 2016). It is therefore possible that the SN activation in our data may reflect the process of salience monitoring and task specification, while the lateral PFC activity in previous studies is more likely to be associated with the regulation component of cognitive control.

There are other possibilities with regard to the findings of the FPN in this study. We examined this issue from a network perspective, which is different from previous studies that investigated the activations of specific brain regions. Furthermore, ICA was employed in this study to dissociate multiple components. Although most component maps from group ICAs are stable, there are still subtle differences in spatial extent of these components each time ICA is applied (Beall and Lowe, 2010). Therefore, the FPN identified by ICA in this study may be subject to the random covariate. Moreover, the trial number is relatively small, resulting to a relatively low SNR. Whether FPN as a network involved in the incentive enhancement of proactive control therefore remains to be established in future studies.

## Limitation

As the order of baseline and reward blocks is fixed, it is possible that the block-based results are confounded by the practice effect. However, this is not necessarily the case here since previous studies did not find practice effect in block-based reward studies (Chiew and Braver, 2013). Moreover, we performed a control analysis and did not find any practice effect in our study. Specifically, a series of paired sample *t*-tests were performed including the comparison between the first and the second block (40 trials) in baseline (RT:* t*_(19)_ = 0.53, *p* = 0.6; error rate: *t*_(19)_ = 0.94, *p* = 0.36), between the first and the last block in baseline (RT:* t*_(19)_ = −0.29, *p* = 0.78; error rate: *t*_(19)_ = 1.29, *p* = 0.21), and between the first half and the last half of trials in the baseline (RT:* t*_(19)_ = 0.09, *p* = 0.93; error rate: *t*_(19)_ = 1.25, *p* = 0.23). Nevertheless, this issue should be further examined in future studies with careful experimental designs.

Note that previous studies commonly explored the reward effect by comparing non-incentive trials in the reward block to baseline trials (sustained effect), and incentive trials in the reward block to non-incentive trials in the reward block (transient; Chiew and Braver, 2013, 2014). In this study, we compared incentive trials in the reward block and baseline trials because the aim of this study was not to dissociate the sustained (block-based) and the transient (trial-based) reward effect. We were interested in the reward facilitation effect of proactive control, and the incentive trials in the reward block have the highest motivation level. Moreover, the number of each trial type was not balanced because of the feature of this reward version AX-CPT (i.e., the number of incentive and non-incentive trials are determined by task performance). This is consistent with the previous studies (close to 1:4 for the number of non-incentive trials compared to that of the baseline trials; Chiew and Braver, 2013, 2014). It would be inappropriate for the neural data to compute the association between network activities and the task phase, as the unbalanced trial number may elicit unstable and inaccurate results (Nasr et al., 2008; Cohen, 2017; Hall et al., 2018). Furthermore, we reanalyzed our data by comparing the reward block to the baseline block (Locke and Braver, 2008), and our results were consistent. A potentially larger trial number or an unequal number of trials for baseline and reward block (e.g., 1:2, to achieve an approximately equal or close trial number of baseline, non-incentive and incentive trials) may be examined in future studies.

We used the same B/Y letters in our study, which may have some influence on our results. To date, AX-CPT has been modified into various versions including emotional AX-CPT (Lamm et al., 2013), No-Go AX-CPT (Gonthier et al., 2016), child-friendly version of the AX-CPT (Kamijo and Masaki, 2016), reward AX-CPT (Chiew and Braver, 2014), and etc. There is also a simplified version of the AX-CPT with four stimuli (two cue and two probe stimuli) that is similar to the task used in this study (Chatham et al., 2009). Moreover, previous studies also used the same B/Y letters to explore reactive and proactive control (e.g., Chang et al., 2017), and the association among monetary incentive, social pressure and reactive/proactive control (e.g., Ličen et al., 2016). However, future studies employing different B/Y letters would be encouraged.

In summary, we explored the brain networks that may be involved in the reward facilitation effect of proactive control. We found that the SN was engaged in the AX-CPT task, and this engagement was positively associated with proactive control particularly in the reward condition. Meanwhile, reward could modulate the AY and BX engagement of the SN in a proactive way. It is possible that this proactive neural modulation supports the enhancement of behavioral proactive control after reward. Our finding suggest that reward may moderate the relationship between task engagement of the SN and proactive control. Findings in this study may provide insights of the neural networks supporting the reward-related promotion of proactive control.

## Author Contributions

AC, GX, HL and LX designed research. LX, LQ and YL performed research. LQ, XC, HL, LZ and YL analyzed data. LQ, XC, LZ and AC wrote the article.

## Conflict of Interest Statement

The authors declare that the research was conducted in the absence of any commercial or financial relationships that could be construed as a potential conflict of interest.

## Footnotes

^1^http://www.fil.ion.ucl.ac.uk/spm

^2^http://icatb.sourceforge.net

^3^http://findlab.stanford.edu/functional_ROIs.html

^4^http://findlab.stanford.edu/functional_ROIs.html
